# Arginase as a Mediator of Diabetic Retinopathy

**DOI:** 10.3389/fimmu.2013.00173

**Published:** 2013-07-03

**Authors:** Chintan Patel, Modesto Rojas, S. Priya Narayanan, Wenbo Zhang, Zhimin Xu, Tahira Lemtalsi, Kanjana Jittiporn, R. William Caldwell, Ruth B. Caldwell

**Affiliations:** ^1^Vision Discovery Institute, Georgia Regents University, Augusta, GA, USA; ^2^Vascular Biology Center, Georgia Regents University, Augusta, GA, USA; ^3^Department of Pharmacology & Toxicology, Georgia Regents University, Augusta, GA, USA; ^4^VA Medical Center, One Freedom Way, Augusta, GA, USA

**Keywords:** arginase, diabetic retinopathy, high glucose, diabetes, oxidative stress, nitric oxide

## Abstract

We have shown previously that diabetes causes increases in retinal arginase activity that are associated with impairment of endothelial cell (EC)-dependent vasodilation and increased formation of the peroxynitrite biomarker nitrotyrosine. Arginase blockade normalizes vasodilation responses and reduces nitrotyrosine formation, suggesting that overactive arginase contributes to diabetic retinopathy by reducing NO and increasing oxidative stress. We tested this hypothesis by studies in streptozotocin-induced diabetic mice and high glucose (HG) treated retinal ECs. Our results show that arginase activity is increased in both diabetic retinas and HG-treated retinal ECs as compared with the controls. Western blot shows that both arginase isoforms are present in retinal vessels and ECs and arginase I is increased in the diabetic vessels and HG-treated retinal ECs. Nitrate/nitrite levels are significantly increased in diabetic retinas, indicating an increase in total NO products. However, levels of nitrite, an indicator of bioavailable NO, are reduced by diabetes. Imaging analysis of NO formation in retinal sections confirmed decreases in NO formation in diabetic retinas. The decrease in NO is accompanied by increased ${\text{O}}_{2}^{.-}$ formation and increased leukocyte attachment in retinal vessels. Studies in knockout mice show that arginase gene deletion enhances NO formation, reduces ${\text{O}}_{2}^{.-}$ and prevents leukostasis in the diabetic retinas. HG treatment of retinal ECs also reduces NO release, increases oxidative stress, increases ICAM-1, and induces EC death. Arginase inhibitor treatment reverses these effects. In conclusion, diabetes- and HG-induced signs of retinal vascular activation and injury are associated with increased arginase activity and expression, decreased bioavailable NO, and increased ${\text{O}}_{2}^{.-}$ formation. Blockade of the arginase pathway prevents these alterations, suggesting a primary role of arginase in the pathophysiological process.

## Introduction

Diabetic retinopathy, a microvascular complication of diabetes, is the leading cause of blindness in adults of working age (1). Diabetes-induced retinal vascular alterations include leukostasis, increased permeability, pericyte loss, appearance of acellular capillaries, and pathological angiogenesis (2, 3). The underlying mechanisms are still unclear. However evidence is accumulating that diabetes-induced retinal vascular dysfunction is associated with hyperglycemia-induced increases in formation of superoxide and peroxynitrite (4–8). Studies have shown that diabetes and high glucose (HG)-induced increases in oxidative and nitrosative stress are accompanied by increases in expression and activity of both endothelial nitric oxide synthase (eNOS) and inducible nitric oxide synthase (iNOS) and that inhibiting NOS activity reduces oxidative and nitrosative stress and prevents early signs of diabetic retinopathy (8–12). However, in spite of the well established link between nitrosative stress and vascular injury in models of diabetes, other work has shown that diabetes impairs ocular hemodynamics by reducing the bioavailability of nitric oxide (NO) (13). NO plays a critical role in maintaining proper tissue blood flow and perfusion, blocking platelet activation and leukocyte adhesion, preventing smooth muscle cell proliferation, and enhancing endothelial cell (EC) survival (14). NO is generated by NO synthase (NOS) from its substrate l-arginine. Acute administration of l-arginine has been shown to increase NO synthesis and restore endothelial-dependent vasodilation in several diseases characterized by vascular dysfunction, including diabetes, hypertension, and heart failure (15, 16), suggesting that decreased l-arginine availability is pivotal to their pathogenesis (17, 18).

Arginase, an enzyme of the urea cycle, uses l-arginine as substrate to produce urea and ornithine. Studies have shown that excessive activity of arginase limits the supply of l-arginine needed for proper NOS function, which will cause uncoupling of the NOS dimer. Uncoupled NOS will use more molecular oxygen to produce superoxide instead of NO. Superoxide will react rapidly with any available NO to produce the highly toxic and proinflammatory oxidant peroxynitrite. This mechanism has been linked to vascular dysfunction in many diseases including diabetes (19, 20). Moreover, angiotensin II, which is upregulated in the diabetic retina, has been shown to increase arginase expression and activity by a mechanism involving activation of P38 MAP kinase/Rho kinase pathway (21).

In a model of endotoxin-induced retinal inflammation, we have shown that elevated arginase activity and expression is correlated with decreases in NO formation, increased cytokine release and retinal inflammation (22). Our studies in rodent models of diabetes have shown that impairment of retinal endothelial-dependent vasodilation is mediated by increases in arginase expression/activity (23). In this study, we hypothesized that increases in arginase expression/activity have a role in diabetes-induced retinal vascular activation/injury via a mechanisms involving decreases in bioavailable NO.

## Materials and Methods

### Treatment of mice

All procedures with animals were performed in accordance with the NIH Guide for the Care and Use of Laboratory Animals and were approved by the institutional animal care and use committee (Animal Welfare Assurance no. A3307-01). Experiments were performed with double knockout mice lacking one copy of arginase I and both copies of II (AI^+/−^AII^−/−^) (24). Deletion of both copies of arginase I is lethal around 10 days postnatal. The knockout mice were provided by Dr. Steven Cederbaum. C57BL6J mice were used as controls. Diabetes was induced by repeated injection (up to four times) of streptozotocin [STZ, 65 mg/kg, dissolved in 0.1 M sodium citrate buffer (pH 4.5), i.p.], once every other day until diabetes was established. Mice with glucose level over 350 mg/dl as determined by a blood glucose meter were considered diabetic. Diabetic and age-matched control mice were used for experiments after 2 months of diabetes.

### Cell culture

Bovine retinal ECs (passages 5–9) were incubated in the medium (M199 + 0.2%FBS + 50 μM l-arginine) containing 5.5 mM d-glucose (NG), 25 mM d-glucose (HG) for 1–3 days. For treatment with the arginase inhibitors [*S*-(2-boronoethyl)-l-cysteine (BEC), 2(*S*)-amino-6-boronohexanoic acid (ABH)], cells were treated with BEC (10 μM) or ABH (100 μM) together with HG.

### Retinal vessel isolation

Retinas were dissected and placed in water on ice for 1 h followed by treatment with Deoxyribonuclease I (116 U/ml, 25–30 min, Worthington Biochemical Corp., Lakewood, NJ, USA). Vessels were rinsed to remove contaminating neurons and glia and then homogenized. Protein was extracted by RIPA lysis buffer (Millipore, Billerica, MA, USA). The supernatant (each containing 10 μg protein) was mixed with 4× loading buffer. After boiling, the samples were analyzed by western blot as described below. Vessel purity was verified by microscopic examination of the vessel preparations. Retinal vessels from six different animals were combined for each determination and five different replications were prepared for both diabetic and control mice.

### Western blot

Retinas or ECs were homogenized in a RIPA Lysis Buffer (Millipore) supplied with phosphatase inhibitor cocktail (Roche), 1 mM phenyl methyl sulfonyl fluoride, and protease inhibitor cocktail (Sigma-Aldrich). Twenty micrograms protein samples were subjected to 10% SDS polyacrylamide gel electrophoresis (SDS-PAGE). Proteins were transferred onto a nitrocellulose membrane and the membrane was blocked (5% milk) and incubated with primary antibodies against arginase I (1:500, Santacruz Biotech), arginase II (1:500, Santacruz Biotech), intercellular adhesion molecule 1 (ICAM-1) (1:500, Santacruz Biotech), actin (1:2000, Sigma-Aldrich), and β-tubulin (1:2000, Sigma-Aldrich), followed by horseradish peroxidase-conjugated secondary antibody (1:2000, GE Healthcare Bio-Sciences). Immunoreactive proteins were detected using the enhanced chemiluminescence (ECL) system (GE Healthcare Bio-Sciences).

### Arginase activity

Retinas or ECs were homogenized in ice-cold lysis buffer (50 mM Tris-HCl, 0.1 mM EDTA, and EGTA, pH 7.5) containing protease inhibitors with a pestle. The homogenate was centrifuged at 14,000 *g* for 20 min and the supernatant was removed for enzyme assay. Arginase activity was assayed as previously described (19). Briefly, the enzyme was activated by heating the lysate at 56°C in 25 mM Tris buffer (pH 7.4) containing 5 mM MnCl_2_. l-Arginine hydrolysis was then conducted by incubating 50 μl of the activated lysate with 50 μl of 0.5 M l-arginine (pH 9.7) at 37°C for 60 min. The reaction was stopped in acid medium. The concentration of urea, which is the end product of l-arginine hydrolysis by arginase, was determined after adding 25 μl of 9% α-isonitrosopropiophenone. Protein concentration in the lysates was determined by a BCA assay (Pierce Biotechnology). Arginase activity was calculated as mmol urea/mg protein and as percent of control.

### Immunofluorescence

Eyes were removed, fixed in 4% paraformaldehyde (overnight, 4°C), washed in PBS and retinas were isolated and cryoprotected in 30% sucrose. Frozen sections (10 μM) were permeabilized in 1% Triton (10 min) and blocked in 10% normal goat serum containing 1% BSA (1 h). Sections were incubated overnight at 4°C in primary antibodies (monoclonal anti-arginase I, 1:200, BD Biosciences; polyclonal anti-arginase II, 1:200, Santacruz Biotechnology; polyclonal CRALBP cellular retinaldehyde binding protein, 1:200, Santacruz Biotechnology) followed by reaction with fluorescein or Texas red conjugated secondary antibodies (Molecular Probes), PBS rinse and mounted with Vectashield (Vector Laboratories).

### Nitrite and nitrate formation

For *in vivo* studies retinas from WT mice with normal glucose (Con), HG (Db), or from AI^+/−^AII^−/−^ mice with HG (Db-Ko) were homogenized in PBS and centrifuged at 14000 rpm for 10 min at 4°C and supernatants were collected. The level of nitrite in the supernatants was analyzed using NO-specific chemiluminescence. In brief, samples containing nitrite were refluxed in glacial acetic acid containing sodium iodide. Nitrite is quantitatively reduced to NO under these conditions, which can be quantified by a chemiluminescence detector after reaction with ozone in a Seivers NO analyzer (NOA 280i, GE Analytical Instruments, Boulder, CO, USA). To measure the total level of nitrite plus nitrate, supernatants were incubated with PBS containing nitrate reductase (0.25 U/ml), NADPH (13 μg/ml), and FAD-Na2 (4 μg/ml) at 30°C for 1 h to reduce nitrate to nitrite. Then the level of nitrite was analyzed using NO-specific chemiluminescence. Protein concentration in the supernatant was determined by BCA assay. The level of nitrite or nitrite plus nitrate was normalized to the protein concentration in the supernatant and calculated as percentage of control. Wild type mice with normal glucose were used as reference. For *in vitro* studies, retinal ECs were treated with 5.5 mM normal glucose (NG), 25 mM HG, or 35 mM glucose for 24 h in M199 medium containing 0.2%FBS and 50 μM l-arginine. Nitrite level in the conditioned media was measured as described in the above procedures for retinal lysates.

### NO formation *in situ*

The NO fluorescent indicator 4,5-diaminofluorescein diacetate (DAF-2 DA, Calbiochem) was used to assess production of NO in retinal tissue sections. In presence of oxygen, DAF-2 reacts with NO to yield the highly fluorescent product triazolofluorescein, which is monitored using excitation and emission wavelengths of 485 and 538 nm, respectively. Unfixed fresh retinal frozen sections from each group were reacted with DAF-2 DA (10 μM, 15 min in the dark at 37°C). One set was pretreated with the NOS inhibitor L-NAME (1 mM). The slides were washed with Hepes solution (10 mM), covered and a series of nine images from each slide were taken by using AxioVision Imaging System (Zeiss). Fluorescence intensity was quantified using MetaMorph Microscopy Image Analysis Software (Molecular Devices).

### Dihydroethidium assay for superoxide formation

To evaluate production of superoxide *in situ* the oxidative fluorescent dye dihydroethidium (DHE) was used as described previously (25, 26). DHE is freely permeable to cells and in the presence of ${\text{O}}_{2}^{.-}$ is oxidized to ethidium bromide which binds to DNA and fluoresces red. The images were analyzed for reaction intensity by using the MetaMorph Image System (Molecular Devices).

### Leukocyte adhesion

Retinal leukostasis was assayed by labeling the adherent leukocytes using Concanavalin A (Vector Laboratories). This method has been described previously (26).

### TUNEL assay

Endothelial cell death was studied using TUNEL (Terminal deoxynucleotidyl transferase dUTP nick end labeling) assay using Fluorescein *in situ* cell death detection kit (Millipore) according to the manufacturer’s protocol. Fluorescent images were taken and the number of TUNEL positive cells was quantified manually.

### Statistical analysis

Data are presented as mean ± SEM. Group differences were evaluated by using one way analysis of variance followed by Tukey’s *post hoc* test for statistical analysis. *p* < 0.05 was considered significant. Animal studies were performed in groups of 6–18 mice. Tissue culture studies were performed in groups of four to eight cultures and each experiment was repeated in at least twice for cells derived from different primary isolates.

## Results

### Arginase activity and expression are increased by diabetes and high glucose

**Figure 1:**
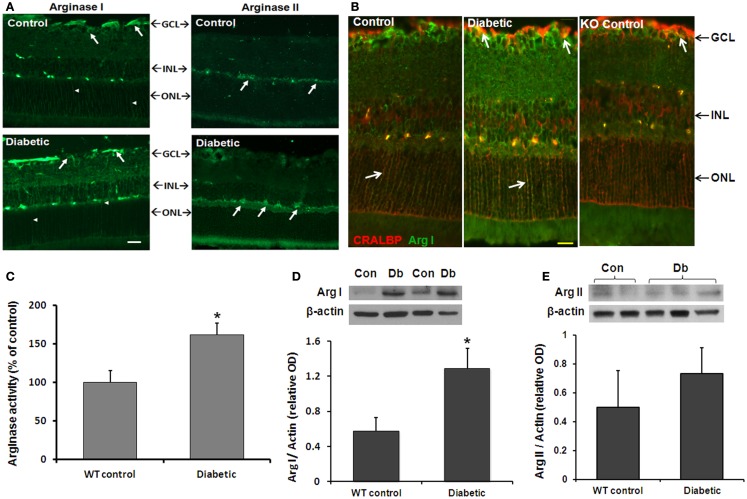
**Diabetes-induced increases in retinal arginase protein and activity levels**. Mice were rendered diabetic with STZ and sacrificed after 2 months. **(A)** Retinal arginase I and II protein distribution were examined by immunofluorescence imaging. Scale bar = 50 μM. **(B)** Double label of arginase I (green) and cellular retinaldehyde binding protein (red) was used to assess arginase I distribution in retinal Müller cells of the wild type and AI^+/−^AII^−/−^ (KO) retinas. Scale bar = 20 μM. **(C)** Arginase activity was determined using an assay for urea formation. Relative levels of arginase I **(D)** and arginase II **(E)** protein expression in retinal vascular cells were determined by Western blot analysis of isolated retinal vessels (*n* = 6–30) (*p < 0.05 compared with the non-diabetic control).

We first examined the effects of diabetes on arginase activity in retinas from STZ-induced diabetic and age-matched control mice (8 weeks). Immunofluorescence analysis showed that both arginase isoforms are expressed in the mouse retina (Figure 1A). The two isoforms differed in their distribution pattern. Arginase I was strongly expressed in cells within the ganglion cell layer and inner nuclear layer and in cells that resemble Müller glia (arrowheads). Localization of arginase I in Müller glia was confirmed by double labeling for arginase I and the Müller cell marker cellular retinaldehyde binding protein (CRALBP; Figure 1B). Retinal vessels in all groups are also labeled due to cross reactivity between mouse vascular proteins and the anti-mouse secondary antibody. Control studies performed in the AI^+/−^AII^−/−^ mice showed low levels of arginase I immunoreactivity, consistent with hemizygous deletion of the arginase I gene. Arginase II was strongly expressed in cells of the inner nuclear layer cells as well as in cells in the nerve fiber and inner plexiform layers. Sections from the AI^+/−^AII^−/−^ mice were negative for arginase II (data not shown). Measurement of arginase activity in retinal tissue extracts using an assay for urea produced by l-arginine hydrolysis confirmed that enzyme activity is significantly increased in the diabetic retinas as compared with the non-diabetic controls (Figure 1C).

The above studies showed that diabetes induces increases in retinal arginase activity and suggested that both isoforms are upregulated. However, there are numerous sources of arginase within the retina. To specifically assess the effects of diabetes in increasing arginase in vascular cells, we isolated retinal vessels from diabetic and control retinas and performed Western blot for arginase I and II. Vessels from three groups of six diabetic and control retinas were isolated by isotonic shock and pooled for Western blot analysis. The results showed that the retinal vessels were positive for both isoforms and that arginase I was increased in the vessels from diabetic mice as compared with the controls (Figure 1D). Levels of arginase II were similar in vessels from the diabetic and control mice (Figure 1E).

**Figure 2:**
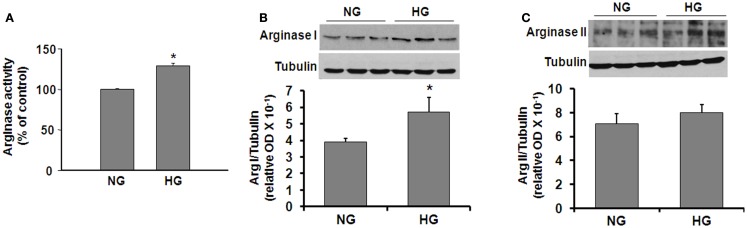
**High glucose-induced increases in arginase protein and activity levels in retinal vascular EC**. Retinal EC (p5–p9) were incubated in medium (M199 + 0.2%FBS + 50 μM l-arginine) containing 5.5 mM d-glucose (NG) or 25 mM d-glucose (HG) for 24 h. **(A)** Arginase activity in cell lysate was determined by arginase activity assay (*p < 0.05 compared with NG, *n* = 4). **(B,C)** Levels of arginase I and II protein were determined by Western blot analysis (*n* = 3–4) and quantified using ImageJ (*p < 0.05 compared with the NG control).

In order to determine more specifically the potential effects of diabetes and hyperglycemia on arginase expression/activity in the vascular endothelium, we performed additional experiments using retinal EC treated with HG media (25 mM glucose). l-Arginine hydrolysis assay showed that HG treatment caused a significant increase in arginase activity (∼30%, Figure 2A). Western blotting showed that both arginase isoforms are expressed in retinal ECs and that arginase I was increased significantly in the ECs treated with HG as compared with the control ECs (Figure 2B). Levels of arginase II were not altered by the HG treatment (Figure 2C).

### Diabetes or high glucose-induced reductions in NO formation are restored by arginase deletion or inhibition

**Figure 3:**
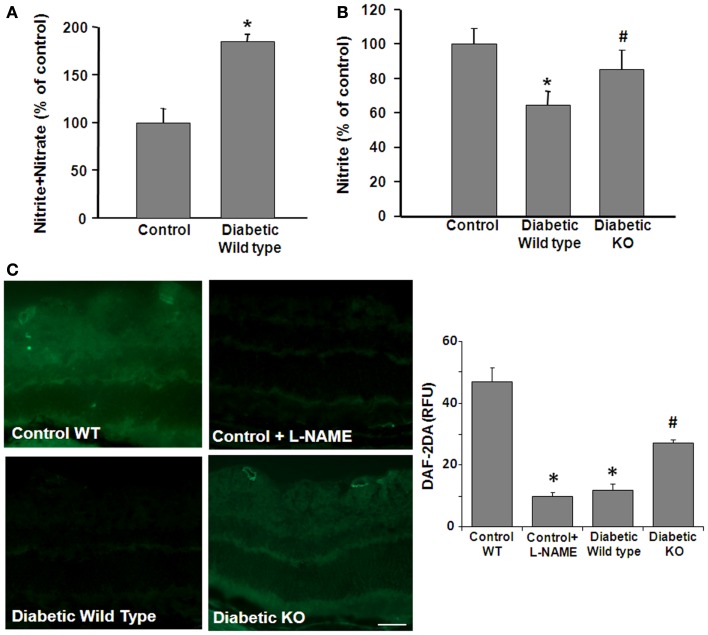
**Prevention of diabetes-induced decrease in bioavailable NO by arginase deletion**. Retinas from normoglycemic wild type controls, wild type diabetic or diabetic knockout mice (KO) were prepared for analysis of NO formation by using an NO analyzer **(A,B)** and by DAF-2-DA histochemistry **(C)**. **(A)** Total NO content in the diabetic retina was significantly increased compared with the control as shown by measurement of nitrate + nitrite levels. **(B)** The level of bioavailable NO in the wild type diabetic retina was significantly reduced compared with the controls as shown by measurement of nitrite levels. The decrease in nitrite levels was blocked in the diabetic KO mice (*p < 0.05, *n* = 4–6). **(C)** NO formation *in situ* was determined by reaction of DAF-2-DA. The DAF-2-DA fluorescent product was significantly diminished in the wildtype diabetic retina as compared with the non-diabetic control. This effect was significantly blunted in the diabetic KO retinas. Pretreatment of the retinal sections with L-NAME (1 mM) markedly reduced formation of the DAF-2-DA product (*p < 0.05 vs. control, #*p* < 0.05 vs. diabetic, *n* = 4–6, scale bar = 50 μM).

We next examined the impact of these diabetes- and HG-induced increases in arginase activity on NO formation. These experiments used an NO analyzer to evaluate bioavailable NO vs. total NO products in control and diabetic retina tissue extracts and HG-treated retinal ECs. In tissue extracts, the total amount of NO is represented by the relative amount of tissue nitrate plus nitrite. Nitrite is the final product of NO auto-oxidation in water or hemoglobin-free media and is an indicator of the amount of bioavailable NO. On the other hand, nitrate formation is promoted in the presence of superoxide. Thus, an increase in nitrate levels serves as an indicator of NOS uncoupling and nitrosative stress. Analysis of total nitrate + nitrite levels showed that STZ-induced diabetes caused a significant increase in the total NO products compared with the controls, whereas nitrite levels were significantly decreased (Figure 3A). To examine the potential role of arginase in these alterations, parallel studies were performed in double knockout mice that lack one copy of the gene for arginase I and both copies of arginase II (AI^+/−^AII^−/−^). The results of this study showed that the diabetes-induced decrease in nitrite was prevented in the AI^+/−^AII^−/−^ mice (Figure 3B), implying the involvement of arginase in the diabetes-induced reductions in bioavailable NO.

The effects of STZ-induced diabetes on tissue distribution of NO were assessed by imaging studies using the NO-specific indicator dye 4,5-diaminofluorescein-2 diacetate (DAF-2 DA). DAF-2 DA reacts with NO to form a green fluorescent product. Fluorescence intensity measurements showed a significant decrease in NO levels in diabetic WT compared to control mice retinas. The DAF-2 DA reaction was predominantly around the blood vessels and in the inner retina and plexiform layers and in the photoreceptor outer segment layer (Figure 3C). The signal was completely blocked by pretreatment with the NOS inhibitor L-NAME indicating the specificity of the reaction for NO. Furthermore, the diabetes-induced decrease in NO was abrogated in the AI^+/−^AII^−/−^ mice, confirming that arginase activity is involved in reducing bioavailable NO levels in the diabetic retina.

**Figure 4:**
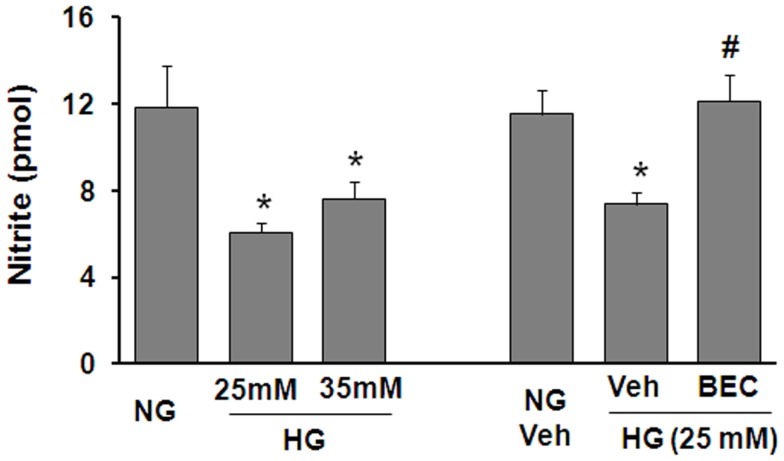
**Prevention of high glucose-induced decreases in bioavailable NO levels in retinal endothelial cells by inhibition of arginase**. Retinal EC (p5–p9) were incubated in medium (M199 + 0.2%FBS + 50 μM l-arginine) containing 5.5 mM d-glucose (NG), 25 mM d-glucose (HG), or 35 mM d-glucose with or without 10 μM BEC or vehicle (Veh, 0.1% saline) for 24 h. The nitrite level in the medium was determined by NO analyzer (*p < 0.05 compared with NG, #*p* < 0.05 vs. Veh,*n* = 8–16).

To further assess the potential involvement of arginase in altering the function of vascular EC NOS during hyperglycemic conditions, we performed *in vitro* studies using retinal ECs. These experiments showed that the HG-induced increase in arginase activity was accompanied by a significant decrease in NO formation as determined by measurement of nitrite in the culture medium using an NO analyzer (Figure 4). The HG-induced decline in NO was blocked by treatment of the cultures with the highly specific arginase inhibitor BEC, suggesting that HG-induced activation of arginase is involved in reducing NO production in retinal ECs. All together, these data support the hypothesis that diabetes reduces nitrite levels in the retina even though total NO products are increased and imply that the reduction in nitrite levels is due to increased arginase activity.

### Diabetes or high glucose-induced increases in oxidative stress levels are diminished by arginase deletion/inhibition

**Figure 5:**
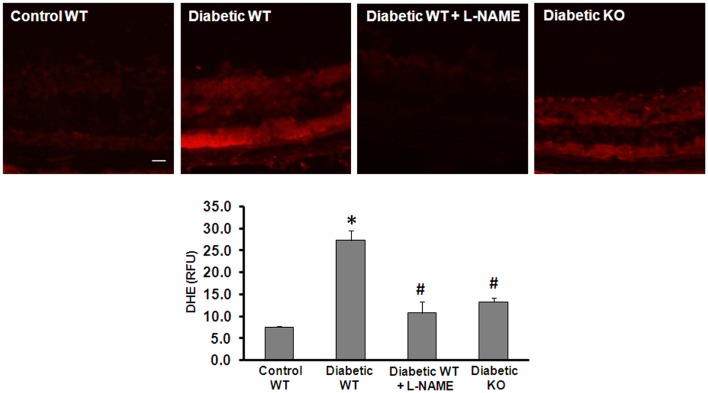
**Prevention of diabetes-induced increase in oxidative stress by arginase deletion**. DHE imaging was performed using flash frozen retinal sections from wild type control, wild type diabetic, and diabetic AI^+/−^AII^−/−^ mice (KO). Diabetic retinas display increased production of ROS as compared with the controls. The increased formation of superoxide was blocked by pretreatment of the sections with L-NAME (1 mM). Arginase KO also significantly blunted the diabetes-induced increase in superoxide formation (*p < 0.05 vs. control, #*p* < 0.05 vs. diabetic wild type, *n* = 6, scale bar = 50 μM).

Increased production of reactive oxygen species (ROS) has been implicated in the pathogenesis of vascular inflammatory diseases involving many organs including the retina (27–29). Uncoupling of NOS serves as an important mechanism of ROS formation in disease. In the present work, we investigated the effect of arginase deletion on ROS production in the diabetic retina. Dihydroethidine (DHE) imaging analysis and quantification of superoxide formation in retinal sections show that superoxide formation was significantly increased in the diabetic retina (Figure 5). This effect was markedly blunted by treatment with the NOS inhibitor L-NAME and in the AI^+/−^AII^−/−^ mice. These results imply that increased arginase activity and uncoupling of NOS are prominently involved in increasing oxidative stress in the diabetic retina. Specificity of the reaction for superoxide was demonstrated by near complete inhibition of the signal by superoxide dismutase (SOD, data not shown).

**Figure 6:**
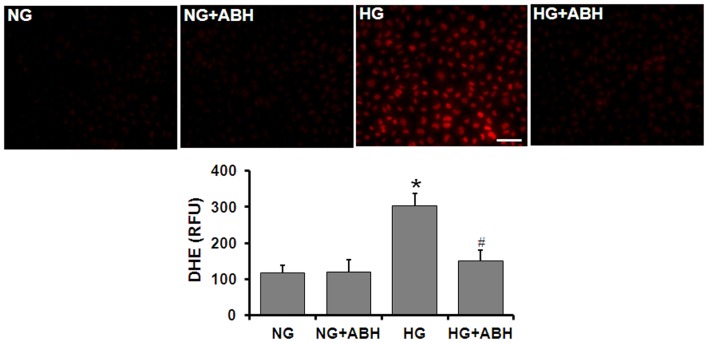
**Prevention of high glucose-induced increase in oxidative stress by arginase inhibition**. Retinal EC (p5–p9) were incubated in medium (M199 + 0.2%FBS + 50 μM l-arginine) containing 5.5 mM d-glucose (NG) or 25 mM d-glucose (HG) for 3 days. DHE imaging shows a significant increase in superoxide formation in the HG-treated ECs. This effect is markedly blunted by treatment with the arginase inhibitor ABH (*p < 0.05 vs. NG, # < 0.05 vs. HG, *n* = 4, scale bar = 50 μM).

Tissue culture studies using DHE imaging of retinal ECs also showed a significant increase in superoxide formation following 3 days of HG treatment (Figure 6). This increase in superoxide formation was significantly inhibited by treatment of the cells with the arginase inhibitor ABH [2(*S*)-amino-6-boronohexanoic acid] or SOD.

### Diabetes-induced increases in leukostasis are blocked by arginase deletion

**Figure 7:**
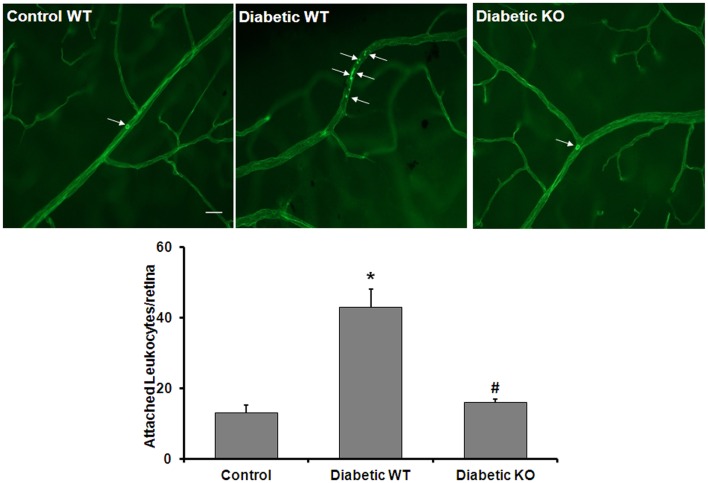
**Prevention of diabetes-induced increase in leukocyte adhesion by arginase deletion**. Wild type controls, wild type diabetic or arginase AI^+/−^AII^−/−^ (KO) diabetic mice were perfused through left ventricle with Concanavalin A to label leukocytes attached to the vascular endothelium. The number of attached leukocytes was significantly increased in the wildtype diabetic mice as compared to non-diabetic controls and arginase KO significantly blunted this effect (*p < 0.05 vs. control, #*p* < 0.05 vs. diabetic wildtype, *n* = 5, scale bar = 100 μM).

An adequate supply of NO is critical for maintaining healthy blood vessels by maintaining appropriate blood flow and inhibiting leukocyte attachment to the vessel walls (30). Therefore, diabetes-induced increases in arginase activity may contribute to vascular injury by increasing leukostasis. To evaluate whether arginase activity plays a role in diabetes-induced retinal vascular activation/injury, we analyzed leukocyte adhesion in the retinal vessels of WT and AI^+/−^AII^−/−^ diabetic and control mice. Leukostasis was assayed by using concanavalin A to label the adherent leukocytes. The data show that the number of adherent leukocytes was increased significantly in the diabetic retinas compared to control retinas and this increase is abrogated by deletion of arginase (Figure 7). This result indicates that increased arginase activity is critically involved in leukocyte-EC attachment in the diabetic retina.

**Figure 8:**
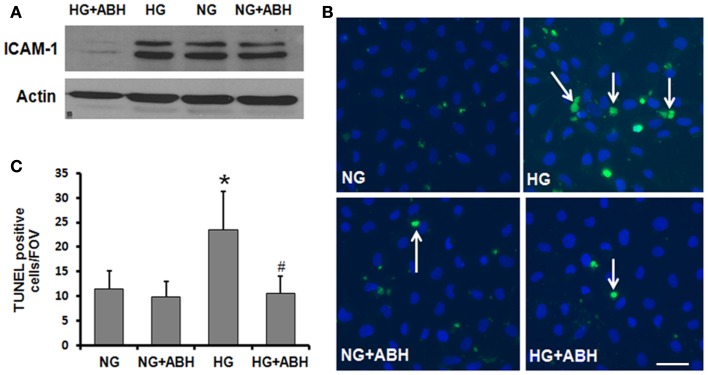
**Prevention of high glucose-induced increase in ICAM-1 levels and cell death in retinal ECs by inhibition of arginase**. Retinal EC (p5–p9) were incubated in medium (M199 + 0.2%FBS + 50 μM l-arginine) containing 5.5 mM d-glucose (NG) or 25 mM d-glucose (HG) for 3 days. **(A)** Western blotting shows that the high glucose-induced increase in ICAM-1 levels is prevented by treatment with the arginase inhibitor ABH. **(B,C)** TUNEL labeling shows that the high glucose-induced increase in cell death is prevented by treatment with ABH (*p < 0.05 vs. NG, # < 0.05 vs. HG, *n* = 4, scale bar = 50 μM).

In order to further assess the potential role of HG-induced increases in arginase activity in causing vascular activation and injury, we examined the effects of HG treatment on expression of ICAM-1. ICAM-1 is present in low concentrations in the membranes of normal ECs, but the concentrations increase under conditions of endothelial activation. ICAM-1 is a ligand for the LFA-1 integrin receptor on leukocytes, which bind to ECs via ICAM-1/LFA-1. Western blotting for ICAM-1 showed that HG treatment of retinal ECs caused upregulation of ICAM-1 and that this effect was abrogated by treatment of the cells with ABH (Figure 8A).

### High glucose-induced increases in cell death are reduced by arginase inhibition

We also investigated the potential impact of arginase activation in retinal EC death under HG conditions. TUNEL assay was performed on cells treated with or without the arginase inhibitor ABH. As shown in Figure 8, cell death was significantly elevated in HG-treated BRECs compared to NG controls. This increase in cell death was prevented by inhibition of arginase, using ABH treatment (Figures 8B,C). These results demonstrate that arginase function is involved in HG-induced EC death.

## Discussion

Excessive activity of the urea cycle enzyme arginase has recently emerged as a critical player involved in the development of numerous vascular disease conditions associated with decreased NO bioavailability and endothelial dysfunction including diabetes, aging, ischemia/reperfusion injury, and hypertension (19, 20, 31–34). Diabetic retinopathy is characterized by progressive vascular damage, beginning with oxidative stress, EC dysfunction, reduced blood flow, and leukostasis (3, 35–37). The pathology often progresses with formation of acellular capillaries, development of diabetic macular edema, and/or vitreoretinal neovascularization. Our previous studies have shown that diabetes-induced increases in arginase activity are involved in hyperglycemia-induced impairment of retinal endothelial-dependent vasorelaxation responses (23). Furthermore, the diabetes-induced increase in arginase activity was accompanied by an increase in peroxynitrite formation which was blocked by treatment with ABH [2(*S*)-amino-6-boronohexanoic acid], a highly specific inhibitor of arginase activity. In this study, we determined the impact of arginase activation on signs of diabetic retinopathy in relation to NO bioavailability, oxidative stress, and retinal injury. Our results showed that diabetes or HG-induced increases in arginase expression and activity in retinal vessels and ECs were associated with decreases in NO, increases in ROS formation, leukocyte adherence to the vessel wall, elevated ICAM-1 expression and death of retinal ECs. These changes were prevented in AI^+/−^AII^−/−^ mice or by treatment with arginase inhibitors, indicating the role of arginase in the pathology.

The effects of diabetes and HG in causing elevation of NOS expression and activity along with increases in oxidative and nitrosative stress in retinas of diabetic animals and retinal ECs are well documented (5, 6, 8, 10, 36). However, this study is the first to show that levels of bioavailable NO are reduced and that this effect is prevented by arginase deletion. Much of the previous work has focused on iNOS as a pathological mediator of retinopathy, emphasizing the damaging effects of iNOS activity and formation of peroxynitrite and other oxidants in relation to vascular EC death and acellular capillary formation in the later stages of diabetic retinopathy (6, 12, 36). Studies have suggested that high levels of NO resulting from upregulation of iNOS expression in retinal glia could be involved in the impairment of retinal blood flow and responses to light during diabetes (38).

Interpretation of NO-mediated toxicity in retinopathy is complicated by the technical difficulty of measuring NO in biological samples. Most studies showing increased “NO levels” in retinal models have used photometric assays to measure total tissue nitrite levels after reduction of nitrate to nitrite. Given that nitrate formation is favored in the presence of excess superoxide (39), the values obtained in such analyses represent relative levels of total NO produced rather than bioavailable NO. So far, little attention has been given to the impact of diabetes on bioavailable NO in retina. However, it is well understood that when the supply of l-arginine needed for NOS activity is limited, the enzyme will become uncoupled and will use molecular oxygen to produce superoxide which combines rapidly with NO to produce the potent inflammatory and toxic oxidant peroxynitrite. Our current results indicate that the amount of bioavailable NO is substantially diminished by HG or diabetes whereas NOS-dependent formation of superoxide is increased, implying that NOS is uncoupled. Furthermore, the decline in NO is blocked by inhibiting or knocking out arginase, consistent with the proposed action of arginase in causing NOS uncoupling. Our study is the first we are aware of to show that arginase is a key player in diabetes/HG-induced decreases in bioavailable NO.

Studies in a variety of peripheral tissues have shown that endothelial dysfunction resulting from impaired NO synthesis involves arginase. Decreased plasma levels of l-arginine have been reported in diabetic animals and patients (40, 41). In diabetic patients, increased arginase activity has been reported in penile vessels associated with erectile dysfunction (42, 43). Experiments with diabetic rat aorta and coronary vessels showed that diabetes-induced impairment of vasorelaxation to acetylcholine was correlated with increased arginase activity. Treatment of bovine coronary ECs with HG also increased arginase activity and diminished NO production, which was normalized by transfection with arginase I siRNA (19). Here we demonstrate for the first time that diabetes and HG treatment significantly increased arginase activity and arginase I protein expression in both retinal vessels and retinal ECs. Immunolocalization studies showed that the two isoforms differ in their tissue distribution. Arginase I was localized to the ganglion cell layer and inner nuclear layer and processes of Muller glial cells. Arginase II was localized to cells of the inner nuclear layer as well as in the nerve fiber and inner plexiform layers. It is possible that both arginase isoforms contribute to increased arginase activity at specific sites within the retina. However the total amount of arginase II protein was similar in vessels isolated from diabetic and control retinas.

In our studies, diabetic mice lacking one copy of arginase I and both copies of arginase II had increased NO levels, less ROS formation and decreased leukostasis as compared with the wildtype diabetic mice, which highlights the important role of arginase expression in diabetes-induced vascular dysfunction. Further work is needed to determine the specific isoform involved. Depending on specific disease conditions, arginase I or arginase II or both may be involved. Arginase I has been associated with endothelial dysfunction in aging, diabetes, and ischemia (19, 32, 44). Arginase II activity has been implicated in atherosclerosis (45) and in retinal neuronal cell degeneration during oxygen-induced retinopathy (46). Others have shown that arginase I is involved in altering NOS function in aging rat aortas (31, 44). Previous studies in coronary ECs have shown that HG-induced increases in arginase activity can be blocked by transfection of arginase I siRNA (19). Our previous studies of retinal vascular function in diabetic mice showed that diabetes-induced retinal vascular dysfunction is reduced in mice lacking one copy of arginase I (23). In order to identify the arginase isoform and cellular sources responsible for specific pathological changes in diabetic retinopathy, additional studies are needed using cell-specific knockout of arginase I and arginase II.

In view of the well established role of iNOS in retinal injury during diabetes (3), the protective effects of arginase blockade in limiting signs of retinopathy may seem paradoxical. However, it is important to note that NO has multiple protective actions, including blocking platelet aggregation and leukocyte adhesion, inhibiting activation of the proinflammatory transcription factor nuclear factor (NF)-κB and modifying lipids to form nitroalkenes, which are anti-inflammatory (47–50). Therefore, under diabetic conditions, inhibition of arginase could limit retinal injury through increased availability of l-arginine for production of NO. Conversely, the beneficial effect of NOS inhibitor treatment in the diabetic retina may be due in part to blockade of uncoupled NOS. A study using vessels from old rats has shown that inhibition of arginase abrogated the aging-induced decrease in eNOS dimer-to-monomer ratio, an indicator of NOS uncoupling (51) suggesting that arginase inhibition restores eNOS coupling and increases bioavailable NO levels. Our present studies indicate that arginase blockade leads to restoration of bioavailable NO and reductions in ROS levels in both the diabetic retina and HG-treated retinal ECs.

We did not investigate the effects of diabetes on NOS expression and activity in the present study because previous work has shown that eNOS, iNOS, and nNOS are upregulated in the diabetic retina (8, 9, 11, 12). HG treatment of retinal ECs also caused an increase in expression of eNOS (10). Furthermore, these effects were accompanied by increases in oxidative stress and peroxynitrite formation and inhibiting NOS or scavenging peroxynitrite reduced oxidative stress and prevented signs of diabetic retinopathy *in vivo* and *in vitro* (8, 10). Results of preliminary studies in which diabetic mice were treated with the arginase inhibitor ABH showed decreased levels of iNOS protein as compared with the vehicle-treated diabetic mice (data not shown). Further study is required to confirm these results and elucidate the underlying mechanisms.

Further study is also needed to determine the mechanisms by which diabetes and HG increase arginase activity. Based on our current studies and previous work of others, multiple factors are likely to affect arginase activity during diabetes. Increased arginase activity is associated with inflammatory cytokines and oxidative stress, both of which are elevated during diabetes (29, 52). Evidence suggests that there is a link between *S*-nitrosylation and enzyme activation in specific inflammatory milieus. It has been shown that iNOS-derived NO directly *S*-nitrosates and activates arginase I in ECs stimulated with IFNγ/LPS (53). Peroxynitrite and H_2_O_2_ have been shown to increase arginase activity in ECs, through PKC mediated activation of RhoA/Rho kinase pathway (54, 55).

In conclusion, our data indicate that arginase is a potential therapeutic target for preserving bioavailability of NO, limiting oxidative stress, and preventing early signs of diabetic retinopathy. Whereas the role of overactive arginase in peripheral vascular dysfunction and injury has been a topic of active research, this is the first time it has been mechanistically linked to retinal vascular injury in diabetes.

## Conflict of Interest Statement

The authors declare that the research was conducted in the absence of any commercial or financial relationships that could be construed as a potential conflict of interest.

## References

[1] NEI The Prevalence of Diabetic Retinopathy Among Adults in the United States (2008). Available from: http://www.nei.nih.gov/eyedata/pbd3.asp

[2] Gardner TW Antonetti DA Barber AJ LaNoue KF Levison SW Diabetic retinopathy: more than meets the eye. Surv Ophthalmol (2002) 47(Suppl 2):S253–62 10.1016/S0039-6257(02)00387-9 12507627

[3] Tang J Kern TS Inflammation in diabetic retinopathy. Prog Retin Eye Res (2011) 30(5):343–58 10.1016/j.preteyeres.2011.05.002 21635964 PMC3433044

[4] Nishikawa T Edelstein D Brownlee M The missing link: a single unifying mechanism for diabetic complications. Kidney Int Suppl (2000) 77:S26–30 10.1046/j.1523-1755.2000.07705.x 10997687

[5] Kern TS Engerman RL Pharmacological inhibition of diabetic retinopathy: aminoguanidine and aspirin. Diabetes (2001) 50(7):1636–42 10.2337/diabetes.50.7.1636 11423486

[6] Du Y Smith MA Miller CM Kern TS Diabetes-induced nitrative stress in the retina, and correction by aminoguanidine. J Neurochem (2002) 80(5):771–9 10.1046/j.0022-3042.2001.00737.x 11948240

[7] Ceriello A New insights on oxidative stress and diabetic complications may lead to a “causal” antioxidant therapy. Diabetes Care (2003) 26(5):1589–96 10.2337/diacare.26.5.1589 12716823

[8] El-Remessy AB Behzadian MA Abou-Mohamed G Franklin T Caldwell RW Caldwell RB Experimental diabetes causes breakdown of the blood-retina barrier by a mechanism involving tyrosine nitration and increases in expression of vascular endothelial growth factor and urokinase plasminogen activator receptor. Am J Pathol (2003) 162(6):1995–2004 10.1016/S0002-9440(10)64332-5 12759255 PMC1868147

[9] Takeda M Mori F Yoshida A Takamiya A Nakagomi S Sato E Constitutive nitric oxide synthase is associated with retinal vascular permeability in early diabetic rats. Diabetologia (2001) 44(8):1043–50 10.1007/s001250100588 11484083

[10] El-Remessy AB Abou-Mohamed G Caldwell RW Caldwell RB High glucose-induced tyrosine nitration in endothelial cells: role of eNOS uncoupling and aldose reductase activation. Invest Ophthalmol Vis Sci (2003) 44(7):3135–43 10.1167/iovs.02-1022 12824263

[11] Leal EC Manivannan A Hosoya K Terasaki T Cunha-Vaz J Ambrósio AF Inducible nitric oxide synthase isoform is a key mediator of leukostasis and blood-retinal barrier breakdown in diabetic retinopathy. Invest Ophthalmol Vis Sci (2007) 48(11):5257–65 10.1167/iovs.07-0112 17962481

[12] Zheng L Du Y Miller C Gubitosi-Klug RA Ball S Berkowitz BA Critical role of inducible nitric oxide synthase in degeneration of retinal capillaries in mice with streptozotocin-induced diabetes. Diabetologia (2007) 50(9):1987–96 10.1007/s00125-007-0772-3 17583794

[13] Toda N Nakanishi-Toda M Nitric oxide: ocular blood flow, glaucoma, and diabetic retinopathy. Prog Retin Eye Res (2007) 26(3):205–38 10.1016/j.preteyeres.2007.01.004 17337232

[14] Loscalzo J Welch G Nitric oxide and its role in the cardiovascular system. Prog Cardiovasc Dis (1995) 38(2):87–104 10.1016/S0033-0620(05)80001-5 7568906

[15] Pieper GM Peltier BA Amelioration by L-arginine of a dysfunctional arginine/nitric oxide pathway in diabetic endothelium. J Cardiovasc Pharmacol (1995) 25(3):397–403 10.1097/00005344-199503000-00008 7769804

[16] Angulo J Rodriguez-Manas L Peiró C Neira M Marín J Sánchez-Ferrer CF Impairment of nitric oxide-mediated relaxations in anaesthetized autoperfused streptozotocin-induced diabetic rats. Naunyn Schmiedebergs Arch Pharmacol (1998) 358(5):529–37 10.1007/PL00005289 9840421

[17] Cooke JP Singer AH Tsao P Zera P Rowan RA Billingham ME Antiatherogenic effects of L-arginine in the hypercholesterolemic rabbit. J Clin Invest (1992) 90(3):1168–72 10.1172/JCI115937 1522225 PMC329981

[18] Tarry WC Makhoul RG L-arginine improves endothelium-dependent vasorelaxation and reduces intimal hyperplasia after balloon angioplasty. Arterioscler Thromb (1994) 14(6):938–43 10.1161/01.ATV.14.6.938 8199185

[19] Romero MJ Platt DH Tawfik HE Labazi M El-Remessy AB Bartoli M Diabetes-induced coronary vascular dysfunction involves increased arginase activity. Circ Res (2008) 102(1):95–102 10.1161/CIRCRESAHA.107.155028 17967788 PMC2822539

[20] Romero MJ Iddings JA Platt DH Ali MI Cederbaum SD Stepp DW Diabetes-induced vascular dysfunction involves arginase I. Am J Physiol Heart Circ Physiol (2012) 302(1):H159–66 10.1152/ajpheart.00774.2011 22058149 PMC3334242

[21] Shatanawi A Romero MJ Iddings JA Chandra S Umapathy NS Verin AD Angiotensin II-induced vascular endothelial dysfunction through RhoA/Rho kinase/p38 mitogen-activated protein kinase/arginase pathway. Am J Physiol Cell Physiol (2011) 300(5):C1181–92 10.1152/ajpcell.00328.2010 21289285 PMC3093945

[22] Zhang W Baban B Rojas M Tofigh S Virmani SK Patel C Arginase activity mediates retinal inflammation in endotoxin-induced uveitis. Am J Pathol (2009) 175(2):891–902 10.2353/ajpath.2009.081115 19590038 PMC2716983

[23] Elms SC Toque HA Rojas M Xu Z Caldwell RW Caldwell RB The role of arginase I in diabetes-induced retinal vascular dysfunction in mouse and rat models of diabetes. Diabetologia (2013) 56(3):654–62 10.1007/s00125-012-2789-5 23232640 PMC3565067

[24] Deignan JL Livesay JC Yoo PK Goodman SI O’Brien WE Iyer RK Ornithine deficiency in the arginase double knockout mouse. Mol Genet Metab (2006) 89(1–2):87–96 10.1016/j.ymgme.2006.04.007 16753325

[25] Al-Shabrawey M Bartoli M El-Remessy AB Platt DH Matragoon S Behzadian MA Inhibition of NAD(P)H oxidase activity blocks vascular endothelial growth factor overexpression and neovascularization during ischemic retinopathy. Am J Pathol (2005) 167(2):599–607 10.1016/S0002-9440(10)63001-5 16049343 PMC1603550

[26] Al-Shabrawey M Rojas M Sanders T Behzadian A El-Remessy A Bartoli M Role of NADPH oxidase in retinal vascular inflammation. Invest Ophthalmol Vis Sci (2008) 49(7):3239–44 10.1167/iovs.08-1755 18378574 PMC3798055

[27] Ellis EA Guberski DL Somogyi-Mann M Grant MB Increased H2O2, vascular endothelial growth factor and receptors in the retina of the BBZ/Wor diabetic rat. Free Radic Biol Med (2000) 28(1):91–101 10.1016/S0891-5849(99)00216-6 10656295

[28] Inoguchi T Li P Umeda F Yu HY Kakimoto M Imamura M High glucose level and free fatty acid stimulate reactive oxygen species production through protein kinase C – dependent activation of NAD(P)H oxidase in cultured vascular cells. Diabetes (2000) 49(11):1939–45 10.2337/diabetes.49.11.1939 11078463

[29] Hink U Li H Mollnau H Oelze M Matheis E Hartmann M Mechanisms underlying endothelial dysfunction in diabetes mellitus. Circ Res (2001) 88(2):E14–22 10.1161/01.RES.88.2.e14 11157681

[30] Lefer AM Nitric oxide: nature’s naturally occurring leukocyte inhibitor. Circulation (1997) 95(3):553–4 10.1161/01.CIR.95.3.553 9024134

[31] Berkowitz DE White R Li D Minhas KM Cernetich A Kim S Arginase reciprocally regulates nitric oxide synthase activity and contributes to endothelial dysfunction in aging blood vessels. Circulation (2003) 108(16):2000–6 10.1161/01.CIR.0000092948.04444.C7 14517171

[32] Hein TW Zhang C Wang W Chang CI Thengchaisri N Kuo L Ischemia-reperfusion selectively impairs nitric oxide-mediated dilation in coronary arterioles: counteracting role of arginase. FASEB J (2003) 17(15):2328–30 14563685 10.1096/fj.03-0115fje

[33] Zhang C Hein TW Wang W Miller MW Fossum TW McDonald MM Upregulation of vascular arginase in hypertension decreases nitric oxide-mediated dilation of coronary arterioles. Hypertension (2004) 44(6):935–43 10.1161/01.HYP.0000146907.82869.f2 15492130

[34] Durante W Johnson FK Johnson RA Arginase: a critical regulator of nitric oxide synthesis and vascular function. Clin Exp Pharmacol Physiol (2007) 34(9):906–11 10.1111/j.1440-1681.2007.04638.x 17645639 PMC1955221

[35] Clermont AC Bursell SE Retinal blood flow in diabetes. Microcirculation (2007) 14(1):49–61 10.1080/10739680601072164 17365661

[36] Kowluru RA Chan PS Oxidative stress and diabetic retinopathy. Exp Diabetes Res (2007) 2007:43603 10.1155/2007/43603 17641741 PMC1880867

[37] Adamis AP Berman AJ Immunological mechanisms in the pathogenesis of diabetic retinopathy. Semin Immunopathol (2008) 30(2):65–84 10.1007/s00281-008-0111-x 18340447

[38] Mishra A Newman EA Aminoguanidine reverses the loss of functional hyperemia in a rat model of diabetic retinopathy. Front Neuroenergetics (2011) 3:10 10.3389/fnene.2011.00010 22291637 PMC3254063

[39] Chen B Keshive M Deen WM Diffusion and reaction of nitric oxide in suspension cell cultures. Biophys J (1998) 75(2):745–54 10.1016/S0006-3495(98)77564-2 9675176 PMC1299749

[40] Hagenfeldt L Dahlquist G Persson B Plasma amino acids in relation to metabolic control in insulin-dependent diabetic children. Acta Paediatr Scand (1989) 78(2):278–82 10.1111/j.1651-2227.1989.tb11070.x 2929351

[41] Pieper GM Dondlinger LA Plasma and vascular tissue arginine are decreased in diabetes: acute arginine supplementation restores endothelium-dependent relaxation by augmenting cGMP production. J Pharmacol Exp Ther (1997) 283(2):684–91 9353386

[42] Bivalacqua TJ Hellstrom WJ Kadowitz PJ Champion HC Increased expression of arginase II in human diabetic corpus cavernosum: in diabetic-associated erectile dysfunction. Biochem Biophys Res Commun (2001) 283(4):923–7 10.1006/bbrc.2001.4874 11350073

[43] Jiang M Jia L Jiang W Hu X Zhou H Gao X Protein disregulation in red blood cell membranes of type 2 diabetic patients. Biochem Biophys Res Commun (2003) 309(1):196–200 10.1016/S0006-291X(03)01559-6 12943682

[44] White AR Ryoo S Li D Champion HC Steppan J Wang D Knockdown of arginase I restores NO signaling in the vasculature of old rats. Hypertension (2006) 47(2):245–51 10.1161/01.HYP.0000198543.34502.d7 16380531

[45] Ryoo S Gupta G Benjo A Lim HK Camara A Sikka G Endothelial arginase II: a novel target for the treatment of atherosclerosis. Circ Res (2008) 102(8):923–32 10.1161/CIRCRESAHA.107.169573 18309100

[46] Narayanan SP Suwanpradid J Saul A Xu Z Still A Caldwell RW Arginase 2 deletion reduces neuro-glial injury and improves retinal function in a model of retinopathy of prematurity. PLoS One (2011) 6(7):e22460 10.1371/journal.pone.0022460 21811615 PMC3141070

[47] Radomski MW Palmer RM Moncada S Endogenous nitric oxide inhibits human platelet adhesion to vascular endothelium. Lancet (1987) 2(8567):1057–8 10.1016/S0140-6736(87)91481-4 2889967

[48] Kubes P Suzuki M Granger DN Nitric oxide: an endogenous modulator of leukocyte adhesion. Proc Natl Acad Sci U S A (1991) 88(11):4651–5 10.1073/pnas.88.11.4651 1675786 PMC51723

[49] Marshall HE Stamler JS Inhibition of NF-kappa B by S-nitrosylation. Biochemistry (2001) 40(6):1688–93 10.1021/bi002239y 11327828

[50] Baker PR Schopfer FJ O’Donnell VB Freeman BA Convergence of nitric oxide and lipid signaling: anti-inflammatory nitro-fatty acids. Free Radic Biol Med (2009) 46(8):989–1003 19200454 10.1016/j.freeradbiomed.2008.11.021 PMC2761210

[51] Kim JH Bugaj LJ Oh YJ Bivalacqua TJ Ryoo S Soucy KG Arginase inhibition restores NOS coupling and reverses endothelial dysfunction and vascular stiffness in old rats. J Appl Physiol (2009) 107(4):1249–57 10.1152/japplphysiol.91393.2008 19661445 PMC2763842

[52] Helmersson J Vessby B Larsson A Basu S Association of type 2 diabetes with cyclooxygenase-mediated inflammation and oxidative stress in an elderly population. Circulation (2004) 109(14):1729–34 10.1161/01.CIR.0000124718.99562.91 15037525

[53] Dunn J Gutbrod S Webb A Pak A Jandu SK Bhunia A S-nitrosation of arginase 1 requires direct interaction with inducible nitric oxide synthase. Mol Cell Biochem (2011) 355(1–2):83–9 10.1007/s11010-011-0841-2 21533769 PMC3744166

[54] Thengchaisri N Hein TW Wang W Xu X Li Z Fossum TW Upregulation of arginase by H2O2 impairs endothelium-dependent nitric oxide-mediated dilation of coronary arterioles. Arterioscler Thromb Vasc Biol (2006) 26(9):2035–42 10.1161/01.ATV.0000233334.24805.62 16794224

[55] Chandra S Romero MJ Shatanawi A Alkilany AM Caldwell RB Caldwell RW Oxidative species increase arginase activity in endothelial cells through RhoA/Rho kinase pathway. Br J Pharmacol (2012) 156:506–19 10.1111/j.1476-5381.2011.01584.x 21740411 PMC3268202

